# The use of theranostic gadolinium-based nanoprobes to improve radiotherapy efficacy

**DOI:** 10.1259/bjr.20140134

**Published:** 2014-08-07

**Authors:** L Sancey, F Lux, S Kotb, S Roux, S Dufort, A Bianchi, Y Crémillieux, P Fries, J-L Coll, C Rodriguez-Lafrasse, M Janier, M Dutreix, M Barberi-Heyob, F Boschetti, F Denat, C Louis, E Porcel, S Lacombe, G Le Duc, E Deutsch, J-L Perfettini, A Detappe, C Verry, R Berbeco, K T Butterworth, S J McMahon, K M Prise, P Perriat, O Tillement

**Affiliations:** ^1^ Institut Lumière Matière, UMR5306 Université Lyon 1-CNRS, Team FENNEC, Université de Lyon, Villeurbanne Cedex, France; ^2^ Institut UTINAM, UMR 6213 CNRS-UFC, Université de Franche-Comté, Besançon Cedex, France; ^3^ Nano-H SAS, Saint Quentin Fallavier, France; ^4^ Institut National de la Santé et de la Recherche Médicale (INSERM), Unité 823, Institut Albert Bonniot, Grenoble, France; ^5^ Université Joseph Fourier (UJF), Grenoble, France; ^6^ Centre de Résonance Magnétique des Systèmes Biologiques, CNRS UMR5536, Université Bordeaux Segalen, Bordeaux, France; ^7^ Clinic for Diagnostic and Interventional Radiology, Saarland University Medical Center, Homburg, Germany; ^8^ Medical Unit of Molecular Oncology and Transfer, Department of Biochemistry and Molecular Biology, University Hospital of Lyon Sud, Hospices Civils of Lyon, Pierre Bénite, France; ^9^ Institut Curie, Equipe Dutreix, Bat 110, Research Centre, Centre Universitaire, Paris-Orsay, France; ^10^ CRAN, UMR 7039, CNRS, Université de Lorraine, Centre Alexis Vautrin, Brabois, Vandoeuvre-lès-Nancy Cedex, France; ^11^ CheMatech, Dijon, France; ^12^ Institut de Chimie Moléculaire de l’Université de Bourgogne, UMR CNRS 5260, Université de Bourgogne, Dijon Cedex, France; ^13^ Laboratoire des Collisions Atomiques et Moléculaires, UMR 8625, Université Paris-Sud 11, CNRS, Orsay Cedex, France; ^14^ ID17 Biomedical Beamline, European Synchrotron Radiation Facility, Grenoble, France; ^15^ Laboratoire Radiothérapie Moléculaire, INSERM 1030, Institut Gustave Roussy Villejuif Labex, LERMIT, Université Paris-Sud, France; ^16^ Dana-Farber Cancer Institute, Brigham and Womens Hospital and Harvard Medical School, Boston, MA, USA; ^17^ Grenoble Institute of Neuroscience, INSERM U836–UJF–CEA–CHU, La Tronche cedex, France; ^18^ Centre for Cancer Research and Cell Biology, Queen's University Belfast, Belfast, UK; ^19^ INSA-Lyon, MATEIS UMR 5510 CNRS, Villeurbanne Cedex, France

## Abstract

A new efficient type of gadolinium-based theranostic agent (AGuIX®) has recently been developed for MRI-guided radiotherapy (RT). These new particles consist of a polysiloxane network surrounded by a number of gadolinium chelates, usually 10. Owing to their small size (<5 nm), AGuIX typically exhibit biodistributions that are almost ideal for diagnostic and therapeutic purposes. For example, although a significant proportion of these particles accumulate in tumours, the remainder is rapidly eliminated by the renal route. In addition, in the absence of irradiation, the nanoparticles are well tolerated even at very high dose (10 times more than the dose used for mouse treatment). AGuIX particles have been proven to act as efficient radiosensitizers in a large variety of experimental *in vitro* scenarios, including different radioresistant cell lines, irradiation energies and radiation sources (sensitizing enhancement ratio ranging from 1.1 to 2.5). Pre-clinical studies have also demonstrated the impact of these particles on different heterotopic and orthotopic tumours, with both intratumoural or intravenous injection routes. A significant therapeutical effect has been observed in all contexts. Furthermore, MRI monitoring was proven to efficiently aid in determining a RT protocol and assessing tumour evolution following treatment. The usual theoretical models, based on energy attenuation and macroscopic dose enhancement, cannot account for all the results that have been obtained. Only theoretical models, which take into account the Auger electron cascades that occur between the different atoms constituting the particle and the related high radical concentrations in the vicinity of the particle, provide an explanation for the complex cell damage and death observed.

Radiotherapy (RT) is the most commonly used non-surgical cancer therapy, designed to apply ionizing radiation at a sufficiently high cytotoxic dose to kill cells within the tumour tissue.^1^ RT is primarily limited in its ability to deliver therapeutic doses to the target tumour volume whilst minimizing damage to the surrounding healthy tissue.^2^ Numerous solutions have been proposed to overcome this issue, broadly falling into two main categories: (i) implementation of advanced RT techniques enabling intensity-modulated radiation fields [intensity-modulated radiation therapy (IMRT)] in order to more precisely adapt the dose to the tumour target; (ii) development of a new generation of therapeutic agents that sensitize cells to ionizing radiation (radiosensitizers) by improving dose efficacy with their high density and high atomic number (*Z*).^3^ High atomic number compounds may provide further benefit in the clinical setting by improving contrast properties for radiological imaging. This would allow monitoring of the radiosensitizing agent within the tumour. It would also facilitate precise defining of the tumour target to allow radiosensitization without affecting healthy tissue. These types of agents are known as “theranostic” agents.

Classical imaging contrast agents based on iodine for CT and gadolinium complexes for MRI could all potentially prove effective theranostic agents. The use of inorganic nanoparticles for radiosensitization was first demonstrated by Hainfeld et al^4^ using 1.9-nm gold nanoparticles delivered systemically prior to irradiation in mice exhibiting EMT-6 mammary carcinomas. The authors reported 1-year survival in 86% of animals treated under these conditions compared with only 20% in those irradiated without gold particle injection. The interest in researching inorganic nanoparticles for the purposes of radiosensitization stems from the unique properties of these particles. Firstly, their innate high atomic number and density characteristics, lending them higher mass energy absorption coefficients than soft tissues;^5^ secondly, their multimodality offering the potential for theranostic applications, such as obtaining imaging functionality in addition to radiosensitizing properties;^6^ thirdly, their particular morphology that enables tailored biodistribution, with the potential for passive targeting due to the enhanced permeability and retention (EPR) effect.^7^ In recent studies, nanoparticles have been shown to induce a highly heterogeneous energy distribution at the subcellular scale, leading to complex cell damage near the particles.^8^ This could be a key factor in determining overall response.

Despite the efficacy of gold particles as radiosensitizers, gold may not be the only suitable high atomic number theranostic candidate, given the lack of sensitivity afforded by CT classically using gold nanoparticles. The combination of MRI and RT technologies for a single image-guided treatment holds clear potential for improved clinical outcome, as emphasized by the development of new fused instruments combining these two modalities. In this context, gadolinium-based particles appear particularly interesting, since their MRI contrast properties are significantly higher than those of molecular complexes in current use, and they also present a strong and promising radiosensitizing effect.

## MRI AND ITS RELEVANCE IN MODERN RT METHODS

The optimization of radiological imaging is essential for improving target delineation at the diagnosis and RT treatment planning stages. The use of conformal RT with high-precision volume targeting, particularly with the tightly conformal doses produced by IMRT or stereotactic radiosurgery (SRS), makes the accurate determination of targeted volume and critical organs essential. CT imaging is commonly used in three-dimensional RT treatment planning in order to outline tumour volume and critical organs, ensuring the superior spatial accuracy and electron density information required for dose calculation algorithms. Yet, the poor soft-tissue contrast obtained with this method may complicate delineation. MRI offers improved soft-tissue contrast and resolution, rendering it increasingly popular for RT. Variations on imaging parameters, such as proton density and tissue relaxation times, can have a significant impact on the image contrast from soft-tissue structures. This flexibility in varying tissue contrast or signal intensities provides much better characterization of soft tissues than can be achieved with radiography. One disadvantage of MRI could involve its capacity for spatial accuracy, although combining CT with MRI scans may correct this inaccuracy and potentially benefit treatment planning. By combining the two techniques, the complementary information contained in both provides a more accurate definition of both tumour and healthy tissues.

For a more reliable medical diagnosis, paramagnetic contrast agents (*T*_1_ or positive contrast agents) are often administered to patients, with the objective of enhancing the native contrast between different tissues. Compared with CT, contrast-enhanced MRI is even more successful at lesion detection, particularly for small lesions, and boasts several advantages. These include improved soft-tissue contrast, the absence of bone artefacts, fewer partial volume effects and direct multiplanar imaging. Advanced MRI techniques, including spectroscopy, diffusion and diffusion tensor, along with perfusion and functional imaging, offer the added benefit of increased physiological data to supplement the anatomic or structural information provided by conventional MRI.^9^

Of all the imaging techniques available in routine practice, MRI performed with a gadolinium-based contrast agent has been recognized as the gold standard in numerous settings for identifying the number, size, characterization (heterogeneity and necrotic area etc.) and location of metastatic lesions along with the invasion of surrounding tissues and critical structures. Taking the case of intracranial metastasis as an example, SRS is the treatment of choice when metastases detected on imaging are few in number (3–5, maximum) or small in size (<30 mm). This treatment also offers the advantage of being minimally invasive and can be used to treat inaccessible lesions, in contrast to surgical resection.^10,11^ With SRS, the capability of MRI to precisely delineate lesion borders in three dimensions is instrumental in reducing recurrence rates and minimizing radiation necrosis in the surrounding tissue. MRI is not only able to generate pathophysiological and functional data pertaining to the central nervous system but also to the lung,^12,13^ breast,^14,15^ prostate,^16^ and head and neck^17^ regions, offering the added possibility of identifying individuals at risk of developing radiation-induced late effects, in addition to monitoring the efficacy of interventions to prevent or improve them. It is therefore of paramount importance that MRI protocols, including the selection of the appropriate gadolinium-based contrast agent, are optimized in order to ensure accurate lesion imaging.^9^

## GADOLINIUM, A KEY ELEMENT AT THE JUNCTURE BETWEEN IMAGING AND THERAPY

Gadolinium is the most widely used paramagnetic element for MRI-positive contrast agents, favoured for its seven unpaired electrons and relatively long electronic relaxation times. Gadolinium-based contrast agents impact on proton relaxation times in the following manner: protons in the organism produce energy when subjected to radiofrequency, which, when correctly amplified, are transformed into the MR signal. Proton relaxation times are typically characterized by two parameters: *T*_1_ (longitudinal relaxation) and *T*_2_ (transverse relaxation). Gadolinium-containing agents reduce *T*_1_ and *T*_2_ relaxation times, thus causing a change in the signal of the injected structures, such as the vessels, parenchyma or lesions.

In addition to the paramagnetic features of gadolinium ions, which are extremely useful for enhancing MRI contrast, the gadolinium element exhibits other properties that are advantageous to radiosensitization, related to its relatively high atomic number (*Z* = 57). During RT, these high atomic number species undergo inner-shell ionization, where one of the deeply bound electrons is removed with high efficiency, compared with results with the low atomic number species predominantly found in living systems. Several Auger emissions can be produced simultaneously from this single inner-shell ionization process, known as an Auger cascade. As a result, these low-energy electrons deposit their energy locally, and this effect can even be amplified if the high atomic number elements are associated in a small solid particle. This highly localized deposition of energy provides the kind of efficient performance usually associated solely with heavy ion facilities, yet using conventional linear particle accelerators (LINAC). Nevertheless, the physical basis of radiosensitization and the resulting biological mechanisms have recently been reviewed.^8,18^

In recent studies, different strategies have been explored pertaining to the design of gadolinium-containing nano-objects. These consisted of either synthesizing crystalline nanoparticles containing gadolinium (gadolinium oxides,^19^ fluoride,^20^ phosphate^21,22^ and vanadates^23^) or functionalizing different types of nanoparticles using gadolinium chelates or ions, either within the structure or on the surface (with liposomes,^24^ zeolites,^25^ mesoporous silica,^26,27^ quantum dots,^28^ lipid particles,^29^ gold nanoparticles,^30,31^ carbon nanotubes^32,33^ and ultrasmall polysiloxane^34,35^). Yet, out of all the gadolinium-based compounds described so far, while most have been developed for MRI application, very few have been described as radiosensitizers.

The most extensively reported and developed gadolinium-based compound for radiosensitization is motexafin gadolinium (MGd), a porphyrin-like macrocycle that forms highly stable complexes with large metal cations. MGd is a tumour-selective radiation sensitizer detectable by MRI^36^ that is currently subject to Phase III clinical development as an adjuvant to RT for brain tumour management. Although this compound is rapidly cleared from blood and healthy tissue, it remains in brain tumours, resulting in a MGd concentration increase observable in the tumour tissue.^37^ Although MGd does not cross the blood–brain barrier (BBB) in healthy brains, MRI revealed its uptake in the brain tumour tissue of glioblastoma multiform (GBM) patients.^38^ The reported MGd mechanism of action is thought to be less significantly modulated by the high atomic number of gadolinium in MGd, but rather more directly associated with complex biochemical processes. This is accounted for by its sensitization of cells through oxidative stress caused by redox cycling, leading to an enhanced radiation response.^39^ The positive effects of a molecule on radiosensitization and chemosensitization are possibly unrelated to the actual presence of the drug and perhaps linked rather to the result of a late MGd effect on energy metabolism or other cell mechanisms involved in enhancing radiosensitivity. MGd (i) initiates an imbalance in the radical scavenging capability of the targeted cells, with an elevated intracellular reactive oxygen species (ROS) production; (ii) negatively regulates the ability of a cell to eliminate ROS; and (iii) inhibits DNA synthesis and repair processes by suppressing the activity of the enzyme ribonucleotide reductase.^37^

Radiosensitization has also been achieved using molecular contrast agents such as Magnevist® (Bayer Pharma, Berlin, Germany) in gadolinium neutron capture therapy (Gd-NCT), an experimental cancer treatment based on the physical principle that neutron capture with ^155^Gd and ^157^Gd ensures the release of local high-dose radiation, such as *γ*-rays and electrons. While an alpha-enhancement factor of 2.3 was obtained with the application of Magnevist on human SW-1573 cells, researchers have observed that Magnevist could not radioenhance the cells for *γ*-ray irradiation. They thus concluded that Gd-NCT, when using a non-toxic concentration of gadolinium, is effective in inducing cell death and chromosome aberrations in *in vitro* cell cultures following neutron radiation.^40^ Based on these findings, we can therefore deduce that, in *γ*-ray irradiation experiments, the nanostructure of gadolinium, such as its unique molecular or clustering of particles, may play a relevant role.

Chitosan nanoparticles containing gadolinium have also been developed and synthetized for radiosensitization; they have successfully been incorporated into cells *in vitro*,^41^ with higher incorporation as than molecular contrast agents. Following intratumoural (IT) injection in melanoma-bearing mice, thermal neutron irradiation was applied to the tumour site, and the 14-day monitoring of tumour growth revealed tumour growth delay.^42^ Designing radiosensitizing gadolinium nanoparticles in order to monitor their distribution using MRI thus constitutes a real asset. The high resolution available with MRI enables the tumour to be accurately located and helps determine the most suitable time for irradiation when a favourable distribution is observed. Of the different nanomaterials containing gadolinium, ultrasmall gadolinium-polysiloxane particles appear a very attractive option.

## DESCRIPTION AND IMAGING PROPERTIES OF AGuIX NANOPARTICLES

### Nanoparticle synthesis

**Figure 1.**
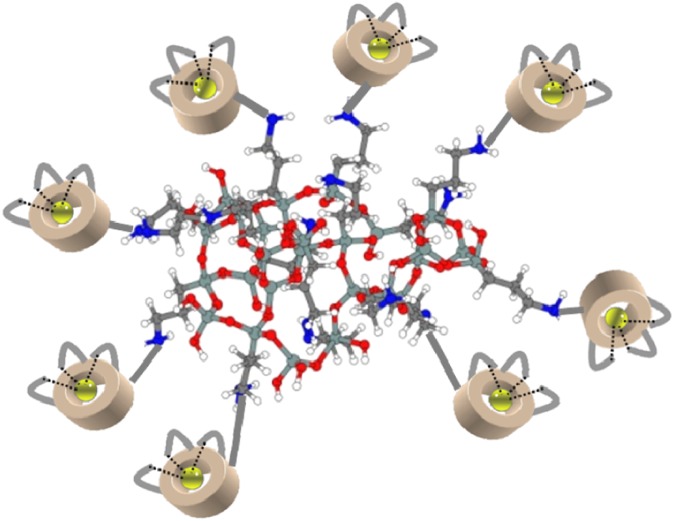
Representation of the AGuIX® nanoparticle. Gadolinium atoms are chelated by 1,4,7,10-tetraazacyclododecane-1,4,7,10-tetraacetic acid derivatives. The polysiloxane matrix is composed mainly of silicon and oxygen. The nanoparticles display a high gadolinium content (15 wt%), sub-5-nm size, approximate mass of 10 kDa, and the following chemical formula Gd_10_Si_40_C_200_N_50_O_150_H_x_.

With the aim of developing nanoparticles for theranostic approaches in cancer RT, our group synthesized ultrasmall gadolinium-based nanoparticles. These were comprised of polysiloxane and surrounded by gadolinium chelates [either diethylenetriaminepentaacetic acid (DTPA) or 1,4,7,10-tetraazacyclododecane-1-glutaric acid-4,7,10-triacetic acid (DOTAGA)] covalently grafted to the polysiloxane inorganic matrix. These nanoparticles exhibited a sub-5-nm size and diameter of approximately 3 nm, which is well suited for renal elimination.^43^ To obtain such small sizes, our laboratory established an original synthesis method.^34,35^ The first generation of AGuIX® nanoparticles was developed with DTPA, an acyclic ligand, already used as a commercial contrast agent named Magnevist.^44^ To further improve thermodynamic and kinetic constants for gadolinium chelation, the same synthesis was performed with DOTAGA, a cyclic ligand. For these nanoparticles, a complexation constant (log*β*_110_) of 24.78 was calculated, almost precisely the same as that of the commercial agent DOTAREM® (Guerbt LLC, Aulnay-sous-Bois, France) (25.58), assessed in the same conditions. There were approximately 10 chelates per nanoparticle, with an approximate mass of 10 kDa (Figure 1). Similar biodistributions and radiosensitizing effects were observed for each of the DTPA or DOTAGA nanoparticles described in this article.

### Biodistribution in healthy animals

**Figure 2.**
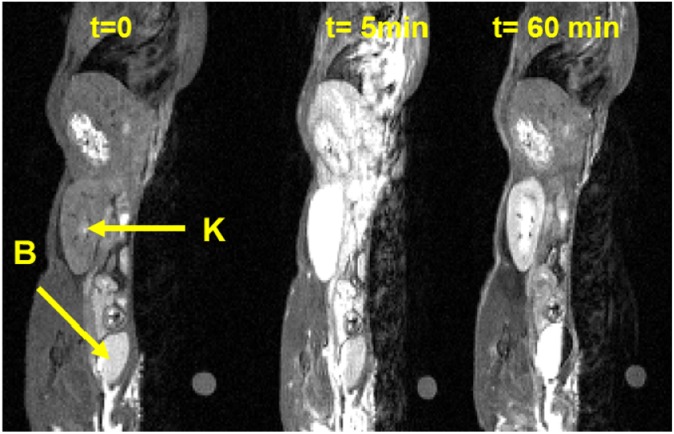
*T*_1_ weighted image of a slice, including a kidney (K) and bladder (B) of a mouse before (*t* = 0), 5 min after and 60 min after intravenous injection of AGuIX® nanoparticles.

AGuIX nanoparticles have been proven as effective MRI-positive contrast agents. The nuclear magnetic relaxation dispersion profile demonstrated that the AGuIX particles display a longitudinal relaxivity (r_1_) two to three times higher than that of DOTAREM, depending on the intensity of the magnetic field [*e.g.* 11.4 mmol^−1^ s^−1^ and a ratio transverse relaxivity (r_2_)/r_1_ of 1.14 at 1.4 T for the AGuIX nanoparticles with DOTAGA]. The nanoparticles were then injected intravenously into healthy mice [80 µl at 40 mM in (Gd^3+^)], with MRI then performed at 7 T. A rapid signal was detected in the kidneys, then in the bladder 5 min after injection, followed by a decrease of the signal due to particle elimination (Figure 2). The residence time of the nanoparticles was approximately double that of the DOTAREM, 13.2 and 6.8 min in mice for AGuIX and DOTAREM, respectively.

**Figure 3.**
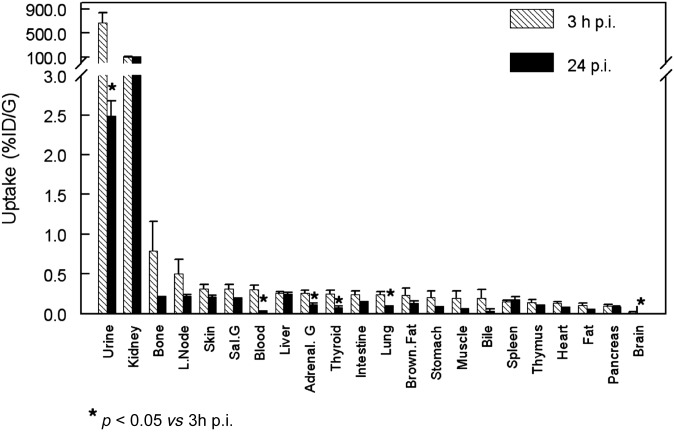
^111^In-labelled AGuIX® biodistribution at 3 and 24 h following intravenous injection in C57Bl6/J mice. G, gland; L, left; ID, injected dose; p.i., post injection; sal, salivary.

By labelling the nanoparticles using ^111^In, we were able to conduct biodistribution studies with a precise quantification of their proportion in each organ following animal sacrifice. Healthy animals were observed exhibiting an uptake in all organs, with the exception of the kidneys and bladder, <0.2% of the injected dose 3 and 24 h following intravenous nanoparticle injection. This confirmed the nanoparticles' renal elimination (Figure 3).^34^

Another specific single photon emission computed tomography study with ^111^In labelling has been performed on brain tumour-bearing animals with longer times following intravenous injection. This study demonstrated that >95% of the nanoparticles were eliminated from the body 18 days after injection.^45^

Brain angiographs were additionally performed in order to compare the contrast obtained using AGuIX with that using DOTAREM at 7 T for the same concentration of gadolinium. A significantly higher contrast was observed for the AGuIX nanoparticles in comparison with DOTAREM, which can be accounted for by the longer residence times observed in the blood vessels for the particles, as well as by their higher longitudinal relaxivity r_1_ (6 mmol^−1^ s^−1^ for the AGuIX and 3 mmol^−1^ s^−1^ for the DOTAREM at 7 T).

**Figure 4.**
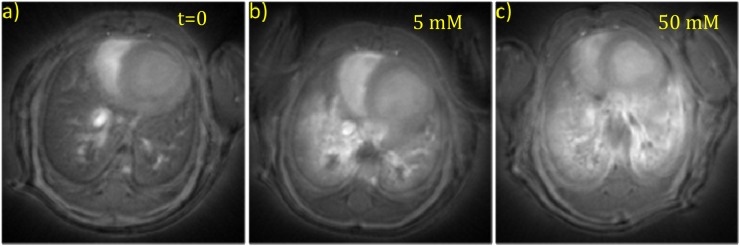
Axial slices of the mouse lungs (a) prior to contrast agent administration (*t* = 0), (b) following administration of 5 mM and (c) 50 mM (of Gd^3+^) of AGuIX® nanoparticles.

Another route of administration was explored in healthy mice, testing the possibility of lung pathology imaging techniques with administration *via* the airways. A clear increase in contrast was observed when conducting an ultrashort echo time (UTE) MRI investigation, a few minutes after administering AGuIX nanoparticles. Different particle concentrations were investigated (Figure 4), with improvements observed up to a maximum concentration of 50 mM in Gd^3+^ (signal enhancement of 266 ± 14%). At higher concentrations, a decrease in signal was observed, probably owing to the *T*_2_ effect. The temporal evolution of signal enhancement recorded in the lungs was fitted by means of a monoexponential, and a lifetime of 149 ± 51 min was determined.^46^ Being small in size, the nanoparticles passed from the lungs into the vascular system and were eliminated by the kidneys and bladder, as expected. In line with previous findings,^35^ no signal was detected in the spleen or liver following intravenous injection. For both differing administration routes, a relatively rapid renal elimination was observed that provided a better contrast in the diseased area and limited the potential toxicity risks.

### Toxicity

Throughout all the synthesis optimizations, formulations and pre-clinical experiments, no real toxicity issues were reported. The chemical composition was based on well-accepted compounds, namely an association of polysiloxane (a silica derivative) and biocompatible gadolinium chelates presenting high stability. Regular animal experiments were performed without any toxicity when using gadolinium concentrations comparable to those in therapeutic use for gadolinium chelate contrast agents (*i.e.* between 5 and 10 mM of gadolinium injected per mouse).

Specific preliminary studies evaluated the maximum tolerated dose pertaining to the nanoparticles in rodents and monkeys. For rats, no adverse effects were observed on repeated weekly nanoparticle injections for 3 weeks, administering concentrations ranging from 250 to 750 mg kg^−1^. For monkeys, repeated injections of nanoparticles, namely 12 in 6 weeks, with concentrations ranging from 100 to 500 mg kg^−1^ were carried out, with no adverse effects observed. These experiments led to an equivalent human dose in the range of 100 mg kg^−1^ being calculated for clinical trial testing.

In addition, an acute toxicological study was conducted on the lungs and kidneys following nanoparticle administration *via* the airways (50 µl of AGuIX at 50 mM in Gd^3+^).^47^ No significant increase in inflammatory cells was observed in the lungs, and there was no pathological change in the alveolar–capillary barrier. Moreover, there was no significant difference in the creatinine levels between the AGuIX and control groups recorded, whereas a significantly elevated creatinine level was noted following treatment by lipopolysaccharide from *Escherichia coli* serotype, as expected. This suggests the absence of any significant nephrotoxicity relating to AGuIX, even in cases of prolonged renal uptake.^47^

### Biodistribution in tumour-bearing animals

Published almost 30 years ago, the studies performed by Maeda^48^ demonstrated the ability of intravenously injected macromolecules or nanoparticles to passively accumulate in tumours. This EPR effect^7^ can at times prove controversial for human application comprising drug delivery with large nanoparticles. In cases using ultrasmall solid particles, the EPR effect may, however, offer advantages, particularly for AGuIX nanoparticles of sizes approaching 10 kDa that exhibit rapid renal elimination.

**Figure 5.**
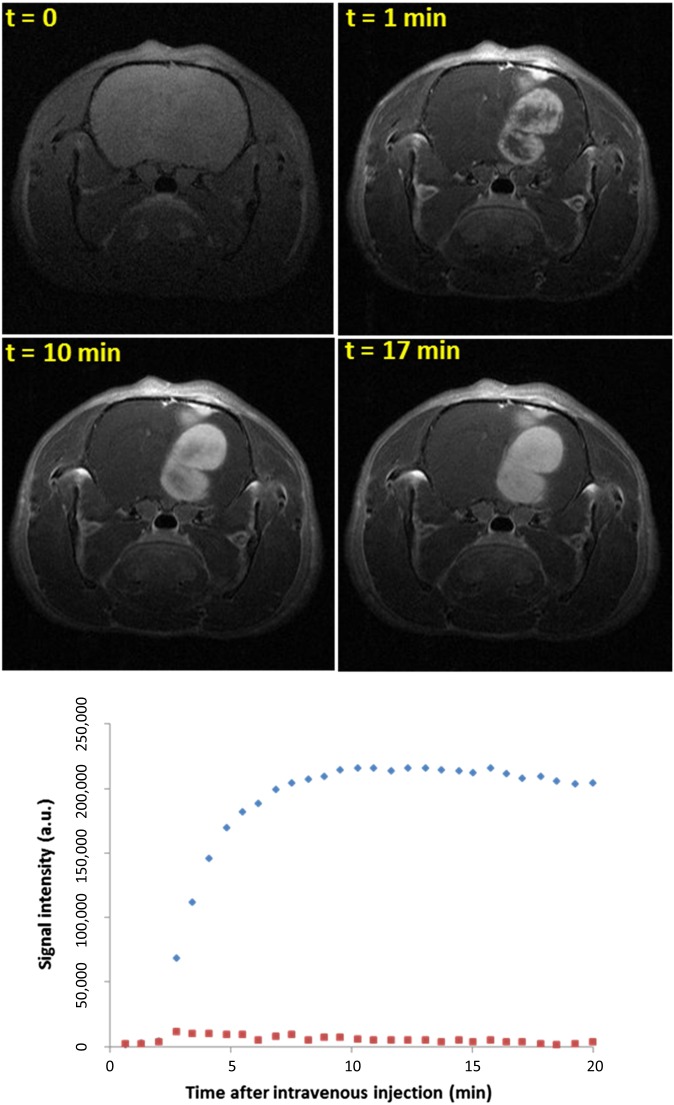
*T*_1_ weighted images of the brain of a 9L gliosacrcoma-bearing rat before and 1, 10 and 17 min after AGuIX® injection. Temporal evolution of the MRI signal in the tumour (diamonds) and in an equivalent surface in tumour tissue in the left hemisphere (squares). a.u., arbitrary unit.

We opted to use orthotopic rather than heterotopic brain tumour models for our analysis of nanoparticle behaviour in a biologically relevant context. In healthy mice, AGuIX nanoparticles do not leak through the BBB, which is often compromised in a pathological brain, thereby allowing drugs to accumulate in the tumour. By injecting AGuIX nanoparticles intravenously into 9L glioma-bearing rats, we were able to increase tumour contrast 1 min following the injection (Figure 5). In the healthy surrounding tissue, the signal was seen to increase slightly a few minutes after the injection before rapidly decreasing. In contrast to this finding, we detected a significant MRI signal enhancement in the tumour, which then plateaued, remaining detectable for up to 24 h post injection. The rapid signal decrease in the healthy zone, combined with the particle retention in the tumour, ensures a large window for performing RT. The best time to activate the nanoparticles with X-rays corresponds to precisely when the concentration is at its highest in the tumour and lowest in the healthy tissue, enabling a high therapeutic effect combined with minimum secondary effects.

In order to provide an example of metastasis visualization, nanoparticles were intravenously injected into rats with hepatic colorectal cancer metastases and visualized by means of a 9.4-T apparatus. This study sought to evidence the adequacy of AGuIX in the most recently developed models of MRI devices, which are designed to operate at greater fields, so as to improve imaging contrast. As we expected, the AGuIX nanoparticles demonstrated better signal-to-noise ratio (SNR), contrast-to-noise ratio (CNR) and lesion enhancement in comparison with DOTAREM when using the same quantities of injected gadolinium (Table 1).[P Fries, 2014, personal communication]

**Table 1.** Evaluation of signal-to-noise ratio (SNR), contrast-to-noise ratio (CNR), and lesion enhancement (LE) in the tumour tissue of the liver in a rat model of hepatic colorectal cancer metastasis following injection of 0.01 mmol kg^−1^ body weight of gadolinium contained in AGuIX® or DOTAREM®

Contrast agent	AGuIX	DOTAREM
SNR	29.6 ± 2.8	18.6 ± 1.2
CNR	6.4 ± 1.2	4.0 ± 0.6
LE	14.9 ± 2.8	3.8 ± 2.7

Data show as mean ± standard deviation.

AGuIX nanoparticles have also been used to detect orthotopic tumours *via* different administration routes. The animals in this experiment were female NMRI immunodeficient mice that had been orthotopically implanted with H358-Luc bioluminescent lung carcinoma cells. Tumour growth was monitored by means of both bioluminescence and CT.

**Figure 6.**
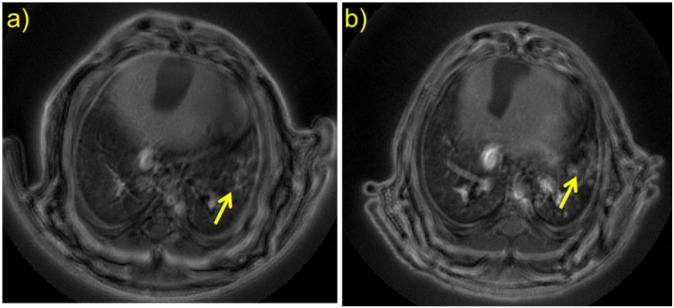
Ultrashort echo time MR images at (a) 35 days and (b) 38 days following tumour implantation (pinpointed by the arrows) and intratracheal administration of 50 µl at 50 mM of AGuIX® nanoparticles. Tumour presence was confirmed by bioluminescence imaging and histology. Adapted with permission from Bianchi et al.^49^

In a recent Proceedings of the National Academy of Sciences publication, intratracheal administration of nanoparticles was compared with intravenous injection of particles at different concentrations, as well as to intravenous injection of DOTAREM (Table 2).^49^ Improved tumour contrast was observed with AGuIX nanoparticles following administration *via* the airways in comparison with intravenous injection (CNR of 15.8 ± 1.3 and 4.8 ± 1.8, respectively), despite the quantity of gadolinium brought by the nanoparticles being four times lower. The marked tumour contrast observed with intratracheal administration cannot be accounted for solely by the EPR effect. One possible explanation for particle accumulation within the tumour is that once the particles reached the alveoli, they were able to directly access the tumour owing to the lack of tissue barrier. The reproducibility of the method was assessed by leaving a 3-day gap between injecting and imaging (Days 35 and 38, respectively, following tumour implantation), which produced the same CNR and signal enhncement results (Figure 6).

**Table 2.** Evaluation of signal enhancement (SE) and contrast-to-noise ratio (CNR) in lung tumours with AGuIX® and DOTAREM® administrated intratracheally and intravenously at different concentrations

Contrast agent and administration route	AGuIX *via* the airways*^a^*	AGuIX *via i.v.**^b^*	AGuIX *via i.v.**^c^*	DOTAREM *via i.v.**^b^*
SE	120.9 ± 30.2	73.9 ± 4.5	31.3 ± 7.8	23.5 ± 5.6
CNR	15.8 ± 1.3	8.3 ± 1.3	4.8 ± 1.8	2.9 ± 2.0

*i.v.*, intravenous injection.

^*a*^ 50 µl at 50.0 mM.

^*b*^ 200 µl at 50.0 mM.

^*c*^ 200 µl at 12.5 mM.

Data shown as mean ± standard deviation.

In summary, MRI experiments have demonstrated that AGuIX nanoparticles enhance contrast much more efficiently than does DOTAREM owing to their higher r_1_ per gadolinium, higher residence time in the body and specific accumulation in tumours, primarily as a result of the EPR effect. MRI visualization of the particles is the first step for personalized medicine. Once their presence has been detected and ideally their concentration quantified using MRI, a radiotherapeutic protocol can be proposed based on the particles' biodistribution measured in the patient.

## STATE-OF-THE-ART METHODS USING NANOPARTICLES AS RADIOSENSITIZERS WITH CONTRAST PROPERTIES

**Figure 7.**
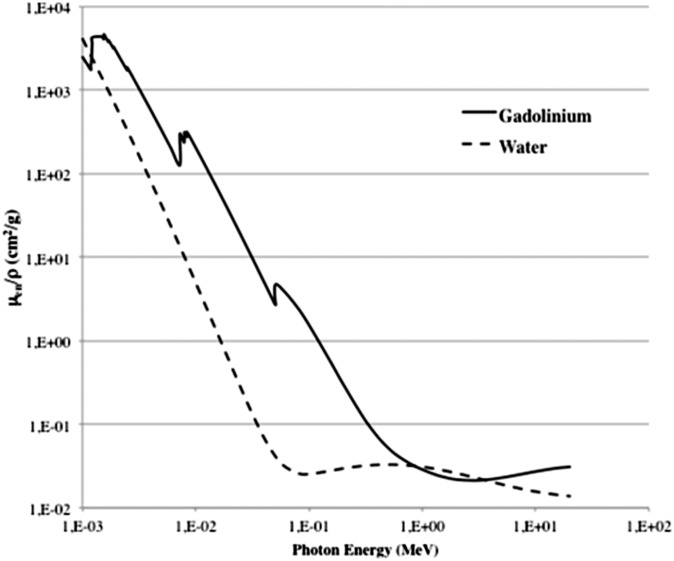
Comparison of photon mass energy absorption coefficients for gadolinium and soft tissues. Adapted from Hubbell and Seltzer.^50^

High atomic number gold (*Z* = 79) and gadolinium (*Z* = 64) particles were used for theranostic applications pertaining to CT and radiosensitization owing to their strong X-ray absorption coefficients resulting in improved contrast over a wide energy range compared with soft tissues (Figure 7).^5,50^

The landmark study by Hainfeld et al^4^ has been succeeded by numerous experimental reports using particles with different physicochemical properties and photon source energies.^8,51^ In parallel, several theoretical models have been developed in the attempt to predict overall sensitization levels based on the mass energy absorption coefficients of gold and soft tissue, gold concentration and source energy. These have taken into account the fact that two major processes govern the interaction between matter and radiation for energies comprised between 1 and 0.9 million electron volts approximately: the Compton effect and the photoelectric effect. At low energies, typically <150 keV, the photoelectric effect dominates. This consists of the absorption of the photon by bound electrons, followed by the ejection of one electron, with re-organization of the other electrons of the atom. As displayed in Figure 7, at this energy range, the mass energy absorption coefficient is significantly higher for gadolinium in comparison with soft tissues and water. At higher energies, particularly in the million electron volts range commonly used for RT, Compton interaction is the effect that predominates. This consists of the inelastic scattering of the incident photon by a bound electron, resulting in its ejection and the re-organization of the electrons of the atom. For this process, there is almost no absorption difference between soft tissues and gadolinium. In these cases, all models have failed to accurately predict the experimental findings. In fact, the observed radiosensitization levels were greater than those predicted by the theory.^3^ As a result, radiosensitization has been attributed to several biological mechanisms, such as oxidative stress, cell cycle and bioavailability.^8^ When using AGuIX gadolinium-based nanoparticles, however, these processes are unlikely to occur. Following these findings, it was later demonstrated that the role of the high atomic number nanoparticles submitted to ionizing radiations was not related to the enhancement of the total dose deposition in the medium, but due rather to the induction of nano-sized spots of intense ionization. This was experimentally described at the molecular scale by Porcel et al,^52^ who demonstrated that platinum nanoparticles induced nano-sized damage as a result of the production of nanoscopic radical clusters in close vicinity to the nanoparticles. Similarly, McMahon et al^53^ reported that ionizing events in gold nanoparticles led to energy depositions equivalent to hundreds of gray units, or even more, in regions within a few hundred nanometres of the nanoparticle. The primary cause of these effects is known to be the Auger cascades occurring in nanoparticles, where single ionizing events led to the emission of multiple low-energy secondary electrons that deposit their energies in short ranges. Owing to the proximity between high atomic number atoms, these cascades are further amplified in nanoparticles, where electrons emitted by one atom can excite neighbouring atoms in their vicinity.

In the following sections of this article, we have reported experimental data concerning the radiosensitizing effect of AGuIX particles, showing that, in addition to being recognized as a better contrast agent than conventional molecular gadolinium complexes for all magnetic fields, AGuIX could also prove effective as a theranostic agent in clinical RT. We have particularly demonstrated the AGuIX effect to be a function of (i) gadolinium concentration, as well as (ii) the nature and (iii) energy of the radiation used in *in vitro* (the *In vitro* experiments: efficacy at low and high energies with small particle concentrations section) and *in vivo* (the *In vivo* experiments section) conditions.

## *IN VITRO* EXPERIMENTS: EFFICACY AT LOW AND HIGH ENERGIES WITH SMALL PARTICLE CONCENTRATIONS

In order to emphasize how significant the radiosensitization process is, we irradiated different tumour cells in the presence of AGuIX nanoparticles. We then analysed the sensitizing enhancement ratios (SER), defined as the survival fraction (SF) ratios for the control cells (irradiation alone) to those of the treated cells (irradiation combined with nanoparticles). Significant SER (ranging from 1.1 to 2.5) were observed, confirming that the particles induce a significant radiosensitizing effect.

Several experiments have been performed testing a large range of conditions, particularly using low gadolinium concentrations (from 0.1 to 1 mM) and kiloelectron volt levels to higher voltages of energy, such as the few million electron volts of energy used in clinical conditions (Table 3). As expected, the nanoparticles exhibited a radiosensitizing effect when subjected to irradiation doses approaching the K-edge of the gadolinium, namely in the kiloelectron volt range where the photoelectric effect predominates. More surprisingly, a similar effect was also observed at higher energies, where the interactions are governed by the Compton diffusion. Our own work was particularly focused on assessing the energy range used for RT in clinical practice. For example, Barberi-Heyob et al demonstrated that AGuIX nanoparticles induced a high radiosensitizing effect on a human glioma cell line labelled U87-MG. The authors found that 6 MV (from 1 to 8 Gy) irradiation of cells incubated with nanoparticles at gadolinium concentrations varying from 0.01 to 0.5 mM led to SER ranging from 1.1 to 1.5 [C Truillet, 2014, personal communication]. The study was completed by Dutreix et al,^56^ concluding that AGuIX nanoparticles primarily induce complex damage. This last experiment evaluated the number of phosphorylated *γ*-H_2_AX histones related to nucleic double-strand DNA breaks. An 84% increase in the number of double-strand breaks was observed following irradiation in the presence of nanoparticles compared with that observed with irradiation alone.

**Table 3.** Radiosensitizing effect of AGuIX® measured on various cell lines

Investigator (team, town)	Radiation/energy	Cell line	NP/incubation time	Biological effect
K. Butterworth (personal communication) (Queen's University, Belfast, UK)	225 keV	Prostate—DU145	From 0.1 to 5.0 mM*^a^*/1 h	1.17 < SF < 2.50
		Glioblastoma—T98G		SF = 1.25
		Prostate—PC3		1.25 < SF < 1.33
R. Berbeco^58^ (Harvard, Boston, MA)	220 kVp X-ray	Cervical carcinoma—HeLa	0.5 mM*^b^*/1 h	SER_4Gy_ = 1.50 DEF = 1.5
C. Rodriguez-Lafrasse^57^ (University Lyon, Lyon, France)	250 kV	Head and neck squamous cell carcinoma—SQ20B	0.4 mM*^a^*/1 h	SF_2_ = 0.60 *vs* 0.72 (SER = 1.20)
			0.6 mM*^a^*/1 h	SF_2_ = 0.35 *vs* 0.72 (SER = 2.00)
		SQ20B cancer stem cells	0.6 mM*^a^*/1 h	SF_2_ = 0.6 *vs* 0.82 (SER = 1.40)
C. Rodriguez-Lafrasse (University Lyon, Lyon, France)^54^	250 kV	Head and neck carcinoma—SQ20B	0.4 mM*^a^*/1 h	SF_2_ = 0.61 *vs* 0.75 (SER = 1.22)
			0.6 mM*^a^*/1 h	SF_2_ = 0.37 *vs* 0.75 (SER = 2.14)
M. Dutreix (Institute Curie, Paris, France)^56^	660 keV	Glioblastoma—U-87MG	0.1 mM/1 h	*γ*-H_2_AX + 80% *vs* irradiation only
			0.5 mM/1 h	
R. Berbeco^56^ (Harvard, Boston, MA)	6 MV	Cervical carcinoma—HeLa	0.5 mM*^b^*/1 h	SER_4Gy_ = 1.30 DEF = 1.2
M. Barberi-Heyob^56^ (CRAN, Nancy, France)	6 MV	Glioblastoma—U-87MG	From 0.01 to 0.50 mM*^b^*/24 h	SER from 1.10 to 1.50
G. Blondiaux (CERI, Orléans, France)	Neutron cyclotron (Orléans, France)	Mouse lymphoma—EL4	From 0.05 to 0.30 mM	Estimated SER_3Gy_ > 2.00
S. Lacombe^59^ (University of Paris-Sud, Orsay, France)	Ions He^2+^ beam (Chiba, Japan)	Chinese hamster ovary carcinoma—CHO	1.0 mM/6 h	SER = 1.14
S. Lacombe^59^ (University of Paris-Sud, Orsay, France)	C^6+^ beam (200 MeV/uma) (Chiba, Japan)	Chinese hamster ovary carcinoma—CHO	1.0 mM/6 h	SER_4Gy_ = 1.50
C. Rodriguez-Lafrasse^57^ (University Lyon, Lyon, France)	C^6+^ (33.6 keV µm^−1^) (Caen, France)	Head and neck carcinoma—SQ20B	0.3 mM*^b^*/1 h	SER = 1.33
			0.6 mM*^a^*/1 h	SER = 1.59

DEF, dose enhancement fraction; NP, nanoparticle; SER, sensitizing enhancement ratio; SF, survival fraction.

Non-human cell lines are indicated.

^*a*^ AGuIX-diethylenetriaminepentaacetic acid.

^*b*^ AGuIX-DOTA.

Another study, performed by Rodriguez et al,^57^ was focused on analysing SQ20B (head and neck squamous cell carcinoma) tumour cells and their cancer stem cell subpopulation, applying 250-kV irradiation following particle incubation at 0.4 and 0.6 mM gadolinium concentration. As with the other cases, the presence of particles during irradiation here caused an SF decrease, combined with a significant increase in the number of double-strand DNA breaks (41% and 53% after exposure at 2 Gy for incubations at 0.4- and 0.6-mM concentration, respectively). Further evidence of the complex damage was provided by the shape of the SF as a function of the deposited dose. Curve fitting was conducted by applying the following linear quadratic equation: SF = exp^−[*α*×D+*β*×D×D]^, where *α* and *β* represent the initial slope (probability of lethal event) and terminal slope (sublethal events) constants, respectively, and D the irradiation dose. For irradiation alone, the fitting curve was characterized by very similar values for *α* and *β* (0.04 and 0.05, respectively). Where AGuIX (0.6 mM in Gd^3+^) was present, the *β*-value was almost unchanged (0.03), whereas *α* was significantly increased up to 0.5. This indicates a high level of direct lethal damage to cells. It is interesting to note that the SF curves with the presence of particles closely resembled those obtained with carbon ion irradiation [SF = exp^−(α×D)^], which is also known to induce complex and irreversible DNA lesions. Lastly, irradiation with ions was conducted, in the presence of nanoparticles, in order to detect synergetic effects. Irradiation by means of He^2+^ or C^6+^ ions in the presence of particles led to SER with the same order of magnitude (Table 3) as that obtained with photon irradiation. This demonstrates that nanoparticle sensitization is universally independent of the radiation type used. ^45,54,55^

In summary, a large variety of *in vitro* experiments have been performed seeking to assess the potential usefulness of gadolinium-based nanoparticles for radiosensitization under different conditions. AGuIX has been proven capable of serving as an efficient radiosensitizer under the following conditions: (i) with different radioresistant cell lines; (ii) at different photon radiation energies ranging from kiloelectron volts to million electron volts; (iii) at very small concentrations in gadolinium (*i.e.* two orders of magnitude smaller than those required by well-established macroscopic dose enhancement models); and (iv) with different types of radiation: photons and fast ions. This increased efficacy is related to the formation of complex and irreversible DNA damage, generated in the vicinity of the particles. Lastly, AGuIX injection can be easily included in traditional RT protocols, as an injection step is often part of examinations using MRI or radiographic CT devices.

## *IN VIVO* EXPERIMENTS

During *in vivo* experiments, different procedures can be applied for particle administration. In order to obtain sufficient radiosensitizing efficacy, the quantity of gadolinium needed in the tumour under irradiation has been estimated at between 1 and 10 µg of gadolinium concentration per gram of living animal. These concentrations have been estimated based on *in vivo* results, and IT administration initially appears more adapted to obtain them, especially for accessible tumours, such as heterotopic or surface tumours.

### Intratumoural administration

**Figure 8.**
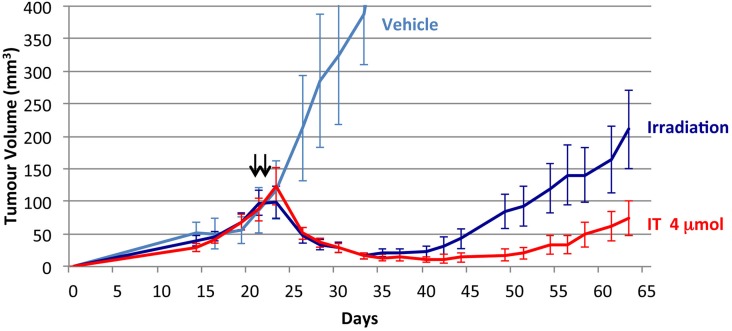
Relative tumour evolution after intratumoral (IT) AGuIX® injection. Tumour evolution without irradiation with only injection of solution without particles (vehicle), 2 × 10 Gy irradiation alone with a delay of 24 h (irradiation) or AGuIX injection (IT 4 μmol, 2 × 4 μmol) followed by 2 × 10 Gy irradiation. Each value represents the mean ± standard error of the mean of tumour volume in mm^3^ (*n* = 8 per group).

IT administration has been evaluated for different types of heterotopic human tumour models,^57^ including radiosensitive melanoma A375. This model was subcutaneously implanted (Figure 8) into the flank of mice, with an injected dose of approximately 4 µmol (0.6 mg). We allowed no delay between injection and irradiation in order to minimize the particle escape that occurs during the treatment.

The treatment was proven to have a positive effect upon tumour development. Tumour size did, in fact, diminish significantly after only two irradiations. Yet after a time, during which the tumour size decreased, some tumour regrowth was observed. The particles' usefulness in this context is particularly supported by the advantage of AGuIX in enabling greater reduction and even near-total suppression of tumour growth. To give a concrete view of the improvement enacted by the presence of particles during irradiation, the tumour volume growth was quantified 25 days following treatment. In this experiment, tumour volume had only increased by 3% under irradiation in the presence of particles, compared with 82% in their absence.

### Intravenous administration

While IT injection may initially appear to be an interesting approach, its clinical application is unfortunately limited to conditions where the tumours lie in the vicinity of the surface of the body. This is particularly the case in head and neck cancers,^57^ which were previously chosen as a relevant example for application. In order to treat deep cancers, as well as the metastases formed over the different disease stages, systemic AGuIX particle administration required investigation, which we conducted *via* intravenous injection. This type of injection requires additional information, in particular a detailed biodistribution analysis, in order to firstly identify the mechanisms of particle elimination and secondly to discriminate between the particle concentration reached in the tumour and that in its healthy surroundings. A strong concentration difference between the two zones was expected owing to the previously described EPR effect.

To evaluate the efficacy of intravenous injections, an orthotopic implantation of 9L gliosarcoma tumour cells into rat brains was performed in order to achieve appropriate tumour size 10 days after implantation. Prior to irradiation, the animals were treated using an intravenous injection of 1.4 ml of AGuIX at 40 mM gadolinium, corresponding to 56 µmol or approximately 10 mg of gadolinium. For an average rat weight of 250 g, this corresponds to an injected dose of 40 µg per gram of rat.

**Figure 9.**
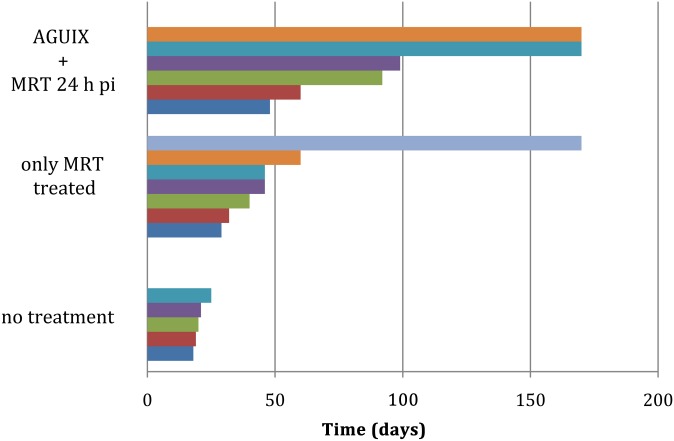
Survival of 9L tumour-bearing rats following tumour cell implantation after no treatment (five individual rats), only irradiated by microbeam radiation therapy (MRT) and irradiated by MRT 24 h after the intravenous nanoparticle injection. p.i., post injection.

RT was performed after a delay of at least 15 min following AGuIX injection, allowing for particle elimination from the surrounding healthy zones by renal clearance and also preventing any potential undesired side effects. With the aid of the EPR effect, this delay does not significantly affect the particle concentration within the tumour, at least for the first few hours following administration. It is this phenomenon, which was closely monitored by MRI, that emphasizes the interest of using theranostic particles for image-guided therapy, particularly considering that irradiation was performed 20 min, 44 min and 24 h post injection (Figure 9—unpublished results).

As shown in Table 4, a significant increase in median survival time (MeST) was observed in both cases following irradiation, compared with untreated animals or those treated only with MRT. The presence of AGuIX during irradiation enabled an increase in survival of the animals by a factor of 4.5 compared with untreated animals and by a factor of 2 compared with irradiated animals. More specifically, MeST was 90.0 and 95.5 days, when combining the intravenous injection of AGuIX and irradiation after a delay of 20 min or 24 h after injection, respectively. These results are clearly accounted for by nanoparticle biodistribution, which was studied in great detail previously.^35^

**Table 4.** Survival results obtained after a microbeam radiation therapy (MRT) experiment

Series	Median survival time (days)	Animals (*n*)	ILS%
Non-irradiated 1	19.0	4	n/a
Irradiated 1	47.0	7	147.0
Irradiated 20 min after injection	90.0	8	373.0
Non-irradiated 2	20.0	5	n/a
Irradiated 2	46.0	7	130.0
Irradiated 24 h after injection	95.5	6	377.5

ILS, increase in life span; MeST, median survival time; n/a, not applicable.

MRT irradiation was performed in cross-firing mode, applying 50-μm-wide microbeams with 200-μm spacing and a 10×10-mm irradiation field centred on the tumour.

The skin entrance dose was set at 400 Gy for the peak and 20 Gy for the valley [ILS = (MeST irradiated−MeST non-irradiated)/MeST irradiated × 100].

Experiments were repeated for the “non-irradiated” and “irradiated” series.

As a result of the EPR effect, and more particularly the retention effect, the gadolinium concentration within the tumour (right brain) was measured at 6 and 4 µg per gram of brain for 20-min and 24-h delays, respectively. In the healthy zone (left brain), it was 5 and 0.5 µg per gram for the two delays, respectively. The extremely small reduction in gadolinium concentration observed in the tumour region emphasizes the efficacy of the EPR effect in its ability to retain the nanoparticles inside the tumour, especially when compared with healthy regions. This could account for the similar efficacy that has been found for irradiations performed both 20 min and 24 h following nanoparticle administration.

**Figure 10.**
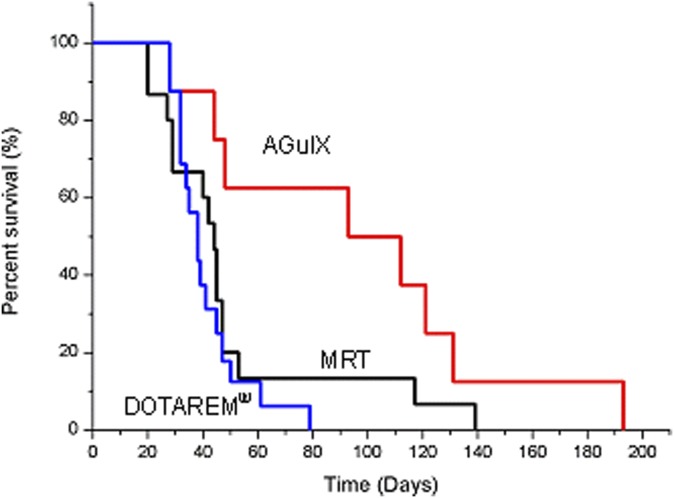
Survival curves of 9L tumour-bearing rats treated only by microbeam radiation therapy (MRT) (MRT, *n* = 15 rats); by MRT 20 min after injection of DOTAREM® (Guerbet, Aulnay-sous-Bois, France) (DOTAREM, *n* = 16 rats; *p* = 0.42 *vs* MRT); and by MRT 20 min after intravenous injection of AGuIX® nanoparticles (AGuIX, *n* = 8 rats; *p* = 0.013 *vs* MRT + DOTAREM and *p* = 0.062 vs MRT). Irradiation was performed 10 days after tumour implantation. MRT irradiation was conducted in cross-firing mode, applying 50-μm-wide microbeams with 200-μm spacing. The skin entrance dose was set at 400 Gy for the peak and 20 Gy for the valley. Statistical analysis was performed using log-rank test.

In addition, a comparison was made between the efficiency of using particles (AGuIX) and molecular complexes such as gadolinium chelates (DOTAREM) (Figure 10) under the same experimental conditions of gadolinium concentration, irradiation, and delay (20 min) between injection and irradiation. The shorter delay of 20 min was chosen in order to maintain a high gadolinium concentration in the tumour during irradiation, especially for the gadolinium molecular contrast agent that is almost entirely cleared from the tumour 24 h after administration. Under these conditions, the survival curve obtained with gadolinium chelates almost exactly correlated with that obtained for irradiation alone: MeST obtained with gadolinium chelates was 38 days, while that for irradiation alone was 44 days. By contrast, the use of nanoparticles produced a MeST of 102.5 days. This survival difference observed between particles and molecular chelates confirms the particle usefulness for radiosensitization. Firstly, this is supported by the better retention observed of particles in the tumour. The second and most significant advantage of using particles is discussed in the Nanoscale dose distribution and radiosensitization section.

### Administration *via* the airways

**Figure 11.**
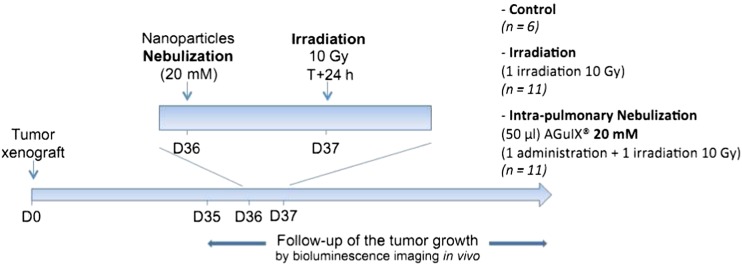
Radiotherapy protocol for orthotopic lung tumour-bearing mice. D, day.

The nanoparticle accumulation observed in lung tumours following nebulization presents an interesting opportunity for RT, particularly considering that there is no accumulation in the healthy zone. In this context, using an orthotopic H358 model of human non-small-cell lung carcinoma grafted by means of intratracheal administration of the cells, we determined that radiosensitization activity was achievable when AGuIX particles (50 µl of approximately 20 mM Gd^3+^) were administered *via* the airways. 1 month after tumour cell implantation, the mice were divided into three groups: control (*n* = 6), irradiation (*n* = 11) and AGuIX administration with irradiation (*n* = 11). Irradiation was performed 24 h after AGuIX administration, consisting of a single 10-Gy dose delivered with a radiation source emitting 200 keV (Figure 11).

Based on survival rates, no statistically significant difference was found between the control and irradiation-only groups (MeSTs of 83 and 77 days, respectively; *p* = 0.926). However, the mice survived much longer when they had received AGuIX particles nebulized 1 day prior to RT. Their MeST was thus extended to 112 days (*p* = 0.028).

In order to optimize RT conditions when using radiosensitizing agents, a large number of parameters must be optimized simultaneously. AGuIX administration is supported by an increasing amount of pre-clinical evidence, yet further optimization of the parameters, including the volume and concentration of gadolinium in the injected solution, the source energy and fractionation schedule, could further increase efficacy. From both a medical and economic viewpoint, a reduced dose requirement for producing the same or an improved biological effect would reduce healthy tissue complications and eventually even treatment costs. High radiosensitization effects have been demonstrated even with gadolinium concentration <1 µg.g^−1^ within the tumour. This is a significant finding, as it proves that even very small gadolinium concentrations are sufficient to induce a major effect on RT efficacy. In particular, it indicates that translation of this method towards early-phase clinical use is a realistic possibility with low quantities of nanoparticles (fewer grams per RT). The gadolinium quantity administered for therapy with AGuIX nanoparticles was, in fact, very similar to that currently injected for MRI, where gadolinium is used in its molecular form as a contrast agent (0.1 mmol kg^−1^). Finally, concentration levels of gadolinium shown to be efficient in RT applications can be compared with those of gold, where gold is used in its particle form for the purpose of sensitizing.^4,54,55^ The gold concentration of the injected particles was 1.9 g kg^−1^ and that in the tumour during irradiation was 7 mg Au g^−1^. These values are over two orders of magnitude greater than the concentration in gadolinium that, in all *in vivo* studies using AGuIX, ranged between 1 and 5 µg Gd g^−1^. RT performed in combination with radiosensitizing particles requires either around 150 g of gold or only a few grams of gadolinium. This demonstrates that gadolinium currently appears to represent a more realistic sensitizer than gold, with the caveats of cell type and radiation source.

## NANOSCALE DOSE DISTRIBUTION AND RADIOSENSITIZATION

As described above, macroscopic dose enhancement predictions based on energy attenuation and gadolinium concentration suggest that no significant enhancement would be seen with the low concentrations used experimentally. Nevertheless, given the relatively high atomic number and multiple electronic shells exhibited by gadolinium, similar Auger cascades to those observed with gold can be seen with this particle, leading to comparable highly localized dose deposits in the vicinity of the nanoparticle.

**Figure 12.**
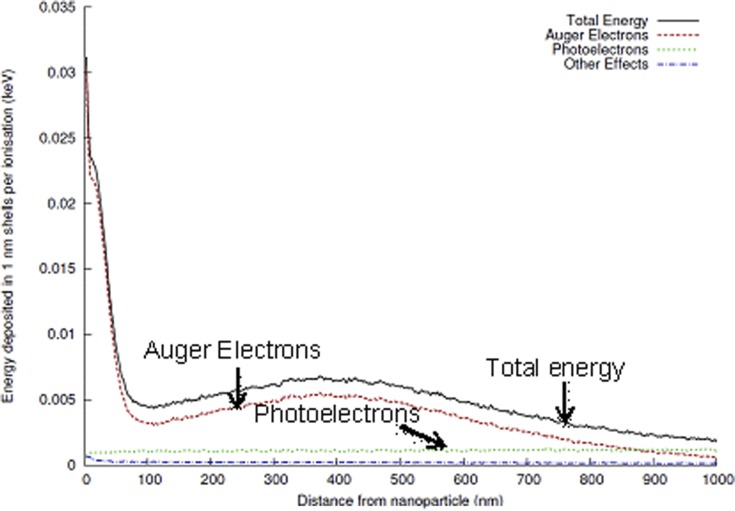
Illustration of nanoscale effects around irradiated AGuIX® gadolinium nanoparticles. The average energy deposited around an AGuIX nanoparticle following an ionizing event by an 80-keV X-ray was calculated using Geant4 (CERN, Meyrin, Switzerland) as a function of distance from the nanoparticle. The primary sources of this energy deposition were Auger electrons (dashed line) and photoelectrons (dotted line), with only a small contribution from other processes (dot-dash line). Owing to the low energy of Auger electrons, they deposit their energy in a highly localized region around the nanoparticle, leading to highly localized doses.

**Figure 13.**
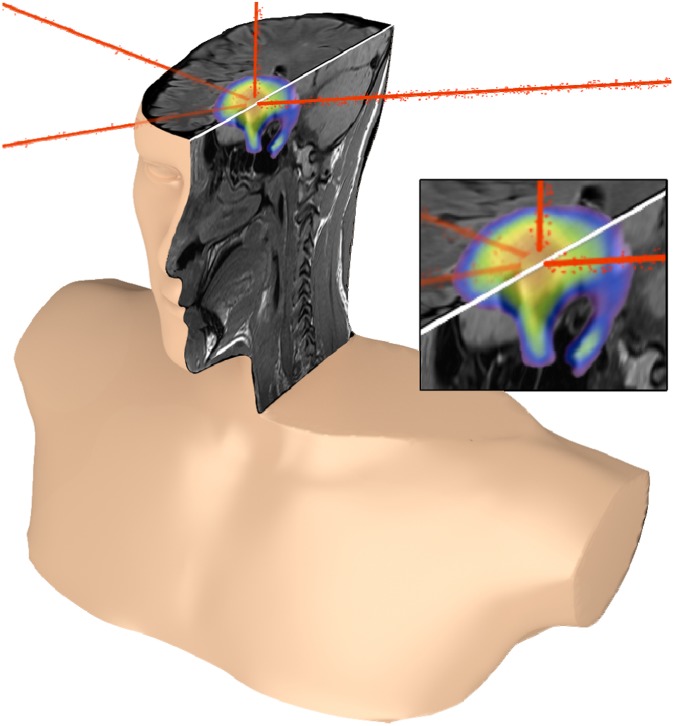
Schematic representation of guided and enhanced radiotherapy with theranostic AGuIX® nanoparticles (Courtesy of T Brichart).

As illustrated, Figure 12 presents a model of the average radial energy deposit in the vicinity of a gadolinium nanoparticle following a single ionizing event by a 80-keV X-ray. Owing to the presence of multiple gadolinium atoms in close proximity, these events display a high probability of triggering an Auger cascade, leading to the production of multiple low-energy electrons depositing large amounts of energy in a short range near the nanoparticle. These particles represent the primary source of energy deposition within a range of <1 μm from the particle (Figure 13).

Owing to the short range of these secondary electrons, these energy depositions correspond to very high doses, namely 2 Gy at 200 nm and almost 10 Gy at 100 nm, along with hundreds to thousands of gray units in the immediate proximity of the particle. These dramatic dose heterogeneities are associated with a range of biological effects, including high production of damaging hydroxyl radicals and other downstream processes. An additional significant factor is that, in the case of ion irradiation, highly localized doses of this form are associated with an increased probability of inducing significant DNA damage and cell death levels.

Based on this observation, an analysis of the impact of using gold nanoparticles in the local effect model suggested that these heterogeneities play a significant role in the greater-than-expected sensitizing effects of gold nanoparticles.^53^ The fact that similar highly localized dose depositions occurred in the presence of gadolinium could, to some extent, explain the discrepancy between simple macroscopic dose predictions and the sensitizing effects we observed.

## CONCLUSION

This review has demonstrated that gadolinium-based nanoparticles hold significant potential as theranostic agents. AGuIX particles, in particular, exhibit unique properties of interest for use not only as an MRI agent for tumour detection but also a radiosensitizer for different radiation types, including photons and fast ions, at different energies, and even at low nanoparticle concentrations. AGuIX particles consist of a polysiloxane network surrounded by gadolinium chelates (generally DTPA or DOTAGA). Owing to their small size (<5 nm) and low mass (approximately 10 kDa), these particles are characterized by biodistributions that are ideal for diagnostic and therapeutic purposes. Following intravenous injection, the particles are rapidly eliminated from the body *via* the renal route and accumulate in tumours up to concentrations reaching 1% of injected dose per gram. This passive accumulation that occurs as a result of the EPR mechanism can be further enhanced by active targeting through peptide functionalization on the surface of the particle.^49^ These particles are always non-toxic, regardless of the administration route chosen (intravenous injection and nebulization). They have also been demonstrated *in vitro* to be efficient radiosensitizers in a large variety of situations, including different radioresistant cell lines and photon radiation energies ranging between kiloelectron volt and million electron volts. This efficiency is related to the formation of a high level of complex and irreversible DNA or cell membrane damage generated in the vicinity of the particles. Finally, the *in vivo* efficacy of AGuIX particles has also been proven in different heterotopic and orthotopic tumours. In the case of 9L gliosarcoma implanted into rats, the therapeutic strategy exploited the theranostic characteristics of AGuIX. Following intravenous injection, the gadolinium concentrations of both the tumour and surrounding healthy tissue were monitored by MRI, enabling RT to be commenced once the concentration in the tumour was sufficiently high, and that in the healthy tissue sufficiently low to avoid adverse effects. A significant increase in lifespan was then obtained for concentrations in the tumour as low as a few micrograms per gram.

The radiosensitizing properties of this compound cannot be described using the concept of macroscopic dose enhancement based on the energy attenuation of the high atomic number metal cations. The effect of gadolinium-based nanoparticles can be attributed to the heterogeneity of dose deposition induced in the medium. Briefly, this phenomenon consists of gadolinium undergoing electronic activation and Auger cascades in a similar way to other high atomic number nanoparticles (gold and platinum), leading to nanoscale dose deposits in the vicinity of the nanoparticle.

This interpretation was supported by our calculation of the average radial energy deposition in the vicinity of gadolinium particles after a single ionizing event. This calculation demonstrated that the low-energy electrons emitted by the nanoparticle deposit their energy in its close vicinity. We found that the presence of several gadolinium ions in the same nanoparticle could even lead to further amplification of the Auger cascade. As a result, the total energy deposition corresponded to extremely high doses being applied close to the particles. This dramatic heterogeneity in dose deposition appears to cause the production of hydroxyl radical clusters and other downstream processes, such as complex biological damage.

The preliminary data collected concerning the toxicity and potential benefits of AGuIX particle use in radiosensitizing applications has indicated that now is an opportune time to commence systematic biological studies, with the objective of initiating early phases first of all in human trials.

## References

[1] (2013). Nanoparticles in radiation therapy: a summary of various approaches to enhance radiosensitization in cancer. *Transl Cancer Res*.

[2] (2008). Radiotherapy in the presence of contrast agents: a general figure of merit and its application to gold nanoparticles. *Phys Med Biol*.

[3] (2008). Variation of strand break yield for plasmid DNA irradiated with high-Z metal nanoparticles. *Radiat Res*.

[4] (2004). The use of gold nanoparticles to enhance radiotherapy in mice. *Phys Med Biol*.

[5] (2013). Radiosensitization by gold nanoparticles: effective at megavoltage energies and potential role of oxidative stress. *Transl Cancer Res*.

[6] (2011). Theranostics: combining imaging and therapy. *Bioconjug Chem*.

[7] (2012). Targeted polymeric therapeutic nanoparticles: design, development and clinical translation. *Chem Soc Rev*.

[8] (2012). Physical basis and biological mechanisms of gold nanoparticle radiosensitization. *Nanoscale*.

[9] (2013). Optimizing contrast-enhanced magnetic resonance imaging characterization of brain metastases: relevance to stereotactic radiosurgery. *Neurosurgery*.

[10] (2013). Neurocognitive assessment following whole brain radiation therapy and radiosurgery for patients with cerebral metastases. *J Neurol Neurosurg Psychiatry*.

[11] (2012). γ knife radiosurgery of brain metastasis from breast cancer. *Prog Neurol Surg*.

[12] (2012). Imaging radiation-induced normal tissue injury. *Radiat Res*.

[13] (2011). MRI-guided tumor tracking in lung cancer radiotherapy. *Phys Med Biol*.

[14] (2013). An investigation of image guidance dose for breast radiotherapy. *J Appl Clin Med Phys*.

[15] (2013). Estimation of heart-position variability in 3D-surface-image-guided deep-inspiration breath-hold radiation therapy for left-sided breast cancer. *Radiother Oncol*.

[16] (2014). MRI target delineation may reduce long-term toxicity after prostate radiotherapy. *Acta Oncol*.

[17] (2013). Multi-scale, multi-modal image integration for image-guided clinical interventions in the head and neck anatomy. *Stud Health Technol Inform*.

[18] (2013). Radiosensitising nanoparticles as novel cancer therapeutics—pipe dream or realistic prospect?. *Clin Oncol (R Coll Radiol)*.

[19] (2013). High-resolution cellular MRI: gadolinium and iron oxide nanoparticles for in-depth dual-cell imaging of engineered tissue constructs. *ACS Nano*.

[20] (2013). Conjugation of NaGdF4 upconverting nanoparticles on silica nanospheres as contrast agents for multi-modality imaging. *Biomaterials*.

[21] (2013). NaGdF4 nanoparticle-based molecular probes for magnetic resonance imaging of intraperitoneal tumor xenografts *in vivo*. *ACS Nano*.

[22] (2013). Synthesis and properties of multifunctional tetragonal Eu: GdPO4 nanocubes for optical and magnetic resonance imaging applications. *Inorg Chem*.

[23] (2013). Fabrication of hollow and porous structured GdVO4: Dy3+ nanospheres as anticancer drug carrier and MRI contrast agent. *Langmuir*.

[24] (2013). A polymeric fastener can easily functionalize liposome surfaces with gadolinium for enhanced magnetic resonance imaging. *ACS Nano*.

[25] (2005). Gadolinium(III)-loaded nanoparticulate zeolites as potential high-field MRI contrast agents: relationship between structure and relaxivity. *Chemistry*.

[26] (2011). Facile synthesis of an up-conversion luminescent and mesoporous Gd2O3 :  Er3+@nSiO2@mSiO2 nanocomposite as a drug carrier. *Nanoscale*.

[27] (2013). Gadolinium(3+)-doped mesoporous silica nanoparticles as a potential magnetic resonance tracer for monitoring the migration of stem cells *in vivo*. *Int J Nanomedicine*.

[28] (2012). Bioconjugation of luminescent silicon quantum dots to gadolinium ions for bioimaging applications. *Nanoscale*.

[29] (2013). Development of a novel lipidic nanoparticle probe using liposomal encapsulated Gd_2_O_3_-DEG for molecular MRI. *J Microencapsul*.

[30] (2013). Gadolinium(III)-gold nanorods for MRI and photoacoustic imaging dual-modality detection of macrophages in atherosclerotic inflammation. *Nanomedicine (Lond)*.

[31] (2013). Nanoamplifiers synthesized from gadolinium and gold nanocomposites for magnetic resonance imaging. *Nanoscale*.

[32] (2009). Biodistribution and ultrastructural localization of single-walled carbon nanohorns determined *in vivo* with embedded Gd2O3 labels. *ACS Nano*.

[33] (2013). Modified Gadonanotubes as a promising novel MRI contrasting agent. *Daru*.

[34] (2013). A top-down synthesis route to ultrasmall multifunctional Gd-based silica nanoparticles for theranostic applications. *Chemistry*.

[35] (2011). Ultrasmall rigid particles as multimodal probes for medical applications. *Angew Chem Int Ed Engl*.

[36] (1996). Gadolinium(III) texaphyrin: a tumour selective radiation sensitizer that is detectable by MRI. *Proc Natl Acad Sci U S A*.

[37] (2003). Response to motexafin gadolinium and ionizing radiation of experimental rat prostate and lung tumors. *Int J Radiat Oncol Biol Phys*.

[38] (2009). Motexafin gadolinium: a novel radiosensitizer for brain tumors. *Expert Opin Pharmacother*.

[39] (2001). Redox cycling by motexafin gadolinium enhances cellular response to ionizing radiation by forming reactive oxygen species. *Int J Radiat Oncol Biol Phys*.

[40] (2006). Gadolinium enhances the sensitivity of SW-1573 cells for thermal neutron irradiation. *Oncol Rep*.

[41] (2002). *In vitro* cellular accumulation of gadolinium incorporated into chitosan nanoparticles designed for neutron-capture therapy of cancer. *Eur J Pharm Biopharm*.

[42] (2000). Gadolinium neutron-capture therapy using novel gadopentetic acid-chitosan complex nanoparticles: *in vivo* growth suppression of experimental melanoma solid tumor. *Cancer Lett*.

[43] (2007). Renal clearance of quantum dots. *Nat Biotechnol*.

[44] (2011). Toward an image-guided microbeam radiation therapy using gadolinium-based nanoparticles. *ACS Nano*.

[45] (2013). Biodistribution of ultra small gadolinium-based nanoparticles as theranostic agent: application to brain tumors. *J Biomater Appl*.

[46] (2013). High resolution contrast enhanced lung MRI in mice using ultra-short echo time radial imaging and intratracheally administrated Gd-based contrast agent. *Magn Reson Med*.

[47] (October 2013). Quantitative biodistribution and pharmacokinetics of multimodal gadolinium-based nanoparticles for lungs using ultrashort TE MRI. *MAGMA*.

[48] (2010). Tumor-selective delivery of macromolecular drugs *via* the EPR effect: background and future prospects. *Bioconjug Chem*.

[49] (2014). Targeting and *in vivo* imaging of non-small-cell lung cancer using nebulized multimodal contrast agents. *Proc Natl Acad Sci U S A*.

[50] (1996). *NIST physical reference data: X-Ray mass attenuation coefficients*.

[51] (2011). Cell-specific radiosensitization by gold nanoparticles at megavoltage radiation energies. *Int J Radiat Oncol Biol Phys*.

[52] (2010). Platinum nanoparticles: a promising material for future cancer therapy?. *Nanotechnology*.

[53] (2011). Nanodosimetric effects of gold nanoparticles in megavoltage radiation therapy. *Radiother Oncol*.

[54] (2010). Gold nanoparticles enhance the radiation therapy of a murine squamous cell carcinoma. *Phys Med Biol*.

[55] (2006). Gold nanoparticles: a new X-ray contrast agent. *Br J Radiol*.

[56] (2011). *In vitro* radiosensitizing effects of ultrasmall gadolinium based particles on tumour cells. *J Nanosci Nanotechnol*.

[57] (2014). Combining gadolinium-based nanoparticles with photon irradiation overcomes radioresistance of head and neck squamous cell carcinoma. *Nanomedicine*.

[58] (2014). Radiation dose enhancement of gadolinium-based AGuIX nanoparticles on HeLa cells. *Nanomedicine*.

[59] (2014). Gadolinium-based nanoparticles to improve the hadrontherapy performances. *Nanomedicine*.

